# Clinical judgement in chest pain: a case report 

**DOI:** 10.1186/s13256-021-02666-z

**Published:** 2021-02-09

**Authors:** Mishita Goel, Shubhkarman Dhillon, Sarwan Kumar, Vesna Tegeltija

**Affiliations:** Department of Internal Medicine, Ascension Providence Rochester Hospital/Wayne State University School of Medicine, 1101 W University Drive, Rochester, MI 48307 USA

**Keywords:** Chest pain, Stress test, Diagnosis, Coronary artery disease

## Abstract

**Background:**

Cardiac stress testing is a validated diagnostic tool to assess symptomatic patients with intermediate pretest probability of coronary artery disease (CAD). However, in some cases, the cardiac stress test may provide inconclusive results and the decision for further workup typically depends on the clinical judgement of the physician. These decisions can greatly affect patient outcomes.

**Case presentation:**

We present an interesting case of a 54-year-old Caucasian male with history of tobacco use and gastroesophageal reflux disease (GERD) who presented with atypical chest pain. He had an asymptomatic electrocardiogram (EKG) stress test with intermediate probability of ischemia. Further workup with coronary computed tomography angiography (CCTA) and cardiac catheterization revealed multivessel CAD requiring a bypass surgery. In this case, the patient only had a history of tobacco use but no other significant comorbidities. He was clinically stable during his hospital stay and his testing was anticipated to be negative. However to complete workup, cardiology recommended anatomical testing with CCTA given the indeterminate EKG stress test results but the results of significant stenosis were surprising with the patient eventually requiring coronary artery bypass grafting (CABG).

**Conclusion:**

As a result of the availability of multiple noninvasive diagnostic tests with almost similar sensitivities for CAD, physicians often face this dilemma of choosing the right test for optimal evaluation of chest pain in patients with intermediate pretest probability of CAD. Optimal test selection requires an individualized patient approach. Our experience with this case emphasizes the role of history taking, clinical judgement, and the risk/benefit ratio in deciding further workup when faced with inconclusive stress test results. Physicians should have a lower threshold for further workup of patients with inconclusive or even negative stress test results because of the diagnostic limitations of the test. Instead, utilizing a different, anatomical test may be more valuable. Specifically, the case established the usefulness of CCTA in cases such as this where other CAD diagnostic testing is indeterminate.

## Background

Heart disease is one of the most commonly encountered medical conditions in the world. Individuals seeking medical help because of chest pain frequently require further testing for heart disease. Cardiac stress testing is a validated diagnostic tool commonly used to assess symptomatic patients with intermediate pretest probability of coronary artery disease (CAD). Baseline electrocardiogram (EKG) findings and ability to exercise are important factors to determine the most appropriate cardiac stress test. Exercise EKG stress test is preferred in patients who have normal baseline EKGs and are able to exercise. Patients found to have positive test results with chest pain usually undergo cardiac catheterization, while those with negative test results are usually considered to have non-cardiac chest pain. In some cases, the cardiac stress test may provide inconclusive results. The decision for further workup typically depends on the clinical judgement of the physician and the results may greatly affect patient outcomes. We present an interesting case of a healthy man who presented with chest pain and had an inconclusive EKG stress test, but further workup was performed and revealed multivessel CAD requiring a bypass surgery.

## Case presentation

A 54-year-old, overweight (BMI 29), Caucasian man with a history of tobacco smoking and gastroesophageal reflux presented to the emergency department with chest pain. He described it as sudden in onset, while he was working on his laptop. Location was substernal, radiating to his left arm and jaw. Initially, the pain was 7/10 in intensity but it improved spontaneously even before he reached the hospital or received any medications. On further probing, he reported that he had experienced intermittent episodes of chest pain for the last 3 weeks but it was mostly exertional and was relieved with rest. The pain was not associated with shortness of breath, diaphoresis, or nausea/vomiting. He denied any fever, chills, cough, abdominal pain, urinary or bowel complaints. He did not have any family history of significant cardiac events.

### Assessment

On presentation, the patient was hemodynamically stable with a blood pressure of 139/85 mmHg and heart rate of 81 beats per minute. His EKG did not show any ischemic changes, no left ventricular hypertrophy, or left bundle branch block. Three sets of serial troponin enzyme were less than 0.010. Lipid panel showed total cholesterol of 235, triglycerides 408, HDL 26, and LDL could not be calculated. His pretest probability of CAD was intermediate on the basis of age and sex. Since the patient was chest pain free since admission and was able to exercise, an exercise treadmill EKG stress test was ordered. The patient achieved 95% of maximum predicted heart rate and 10 METs of exercise with normalization of slight T wave inversions that were seen in leads V2, V3, and V4 at rest. Thus, it was read as maximum asymptomatic stress test with intermediate probability of ischemia. Echocardiogram was obtained which showed normal left ventricular function and no significant valvular or wall motion abnormalities. At this point, cardiology was consulted to evaluate the patient and they recommended coronary computed tomography angiography (CCTA) for further risk stratification.

### Diagnosis and management

**Fig. 1:**
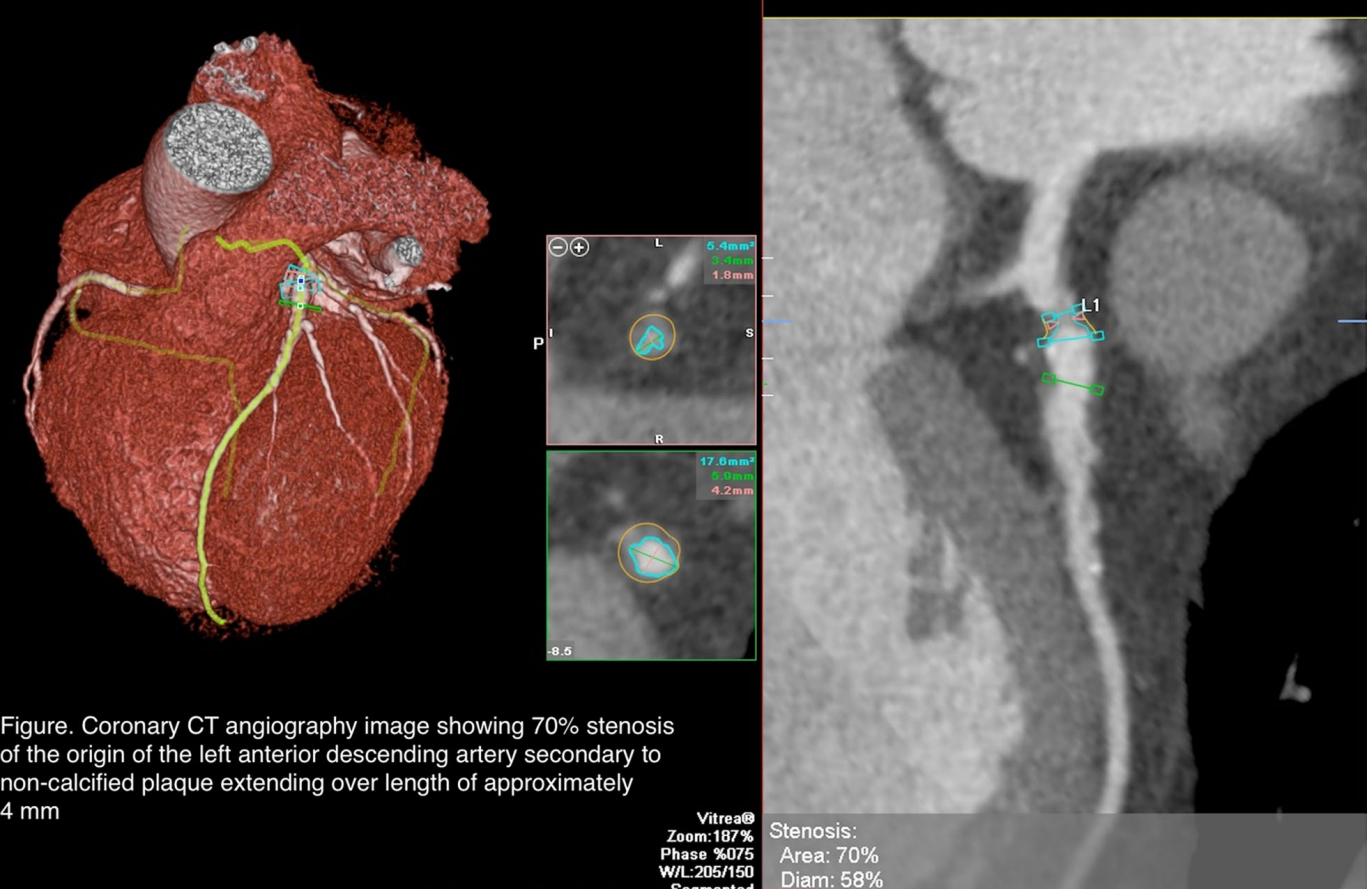
Coronary computed tomography angiography image showing 70% stenosis of the origin of the left anterior descending artery to secondary to non-calcified plaque extending over a length of approximately 4 mm

CCTA results showed approximate 70% stenosis of the origin of the left anterior descending artery (LAD) secondary to noncalcified plaque extending over a length of approximately 4 mm (Fig. 1), approximate 40–50% stenosis of the proximal ramus intermedius branch secondary to mixed calcified and noncalcified plaque and scattered calcified and noncalcified plaque along the circumflex and obtuse marginal branches with 30–40% luminal diameter stenosis. Fractional flow reserve–computed tomography (FFR-CT) revealed a high likelihood of flow-limiting stenosis with a value of less than 0.5 secondary to the significant stenosis at the origin of the LAD and a low likelihood of flow-limiting stenosis in the left circumflex, ramus intermedius, and right coronary arteries.

The patient was then taken for cardiac catheterization which showed a 95% stenotic lesion of LAD with partial perfusion (TIMI grade 2 flow) giving rise to diagonal 1, which has an ostial and proximal 70% stenosis; ramus intermedius with proximal 70% segmental stenosis; circumflex, nondominant vessel, which has mild disease in proximal distal segments, giving rise to obtuse marginal 1, which has proximal 70% stenosis. Cardiothoracic surgery was consulted and the patient underwent bypass graft surgery.

### Outcome and follow-up

The patient did well after the surgery. He stayed in the hospital for 4 days post-op without any complaints and was discharged home in stable condition. A referral to home care was made to provide for monitoring of the patient's progress and detection of any complications during the immediate post-op period. Cardiac rehabilitation referral was also provided and the patient was instructed to follow up with a cardiologist and cardiothoracic surgeon.

## Discussion

Cardiac stress testing is usually performed for diagnosis of CAD in patients with intermediate pretest probability of CAD. Appropriate history, physical examination, and baseline EKG findings are crucial in determining the most appropriate and cost-effective stress test for these patients. According to the American College of Cardiology (ACC)/American Heart Association (AHA) guidelines, exercise stress EKG is recommended as an initial diagnostic test among patients at intermediate pretest risk who are able to exercise and who have an interpretable resting EKG [1]. In the presence of baseline EKG abnormalities, including ST depression greater than 1 mm, left ventricular hypertrophy, left bundle branch block, paced rhythm or pre-excitation, functional tests with imaging or anatomical tests including CCTA are preferred. Studies have shown that exercise EKG test is adequate for risk stratification of cardiac events which are found to be very low in patients with a negative EKG stress test [2].

Our case describes a commonly encountered scenario of a patient with few risk factors for CAD who presented to the hospital with chest pain and requires further diagnostic testing for CAD. Treadmill EKG is one of the most utilized CAD testing methods in our practice and the results guide further management of patients presenting with chest pain. A meta-analysis including 22 years of research revealed the pooled sensitivity of EKG stress test in detecting CAD to be 68% and specificity to be 77% [1]. Despite this, EKG stress tests continue to be one of the most commonly used and trusted tools in our clinical practice.

A cohort study comparing usefulness of dipyridamole echocardiography, dobutamine-atropine echocardiography, and exercise stress testing revealed similar sensitivity for diagnosis of CAD in patients presenting with chest pain [3]. In fact even in cases of multivessel CAD, studies have shown similar sensitivity of all three tests [4]. Studies have shown low prevalence of significant ischemia and CAD mortality in patients achieving more than 10 METs on exercise stress test [5]. In a 2014 randomized controlled trial, all cause mortality was found to be similar in patients with suspected CAD and normal resting EKG who underwent EKG stress test with imaging compared to those without imaging [6].

Physicians seldom see reports of indeterminate stress test results which is when they depend on expert opinion for further evaluation. In this case, the patient was an overweight 54-year-old man who had a history of tobacco use but no other significant comorbidities were known. He was clinically stable during his hospital stay. We anticipated his testing would be negative. To complete workup, cardiology recommended anatomical testing with CCTA given the indeterminate EKG stress test results and this was performed immediately. The results of significant stenosis were surprising to the care team. CCTA is a relatively newer non-invasive anatomical test that has a high diagnostic accuracy to identify the presence of coronary plaques and stenosis. Since it can also determine the extent of stenosis, it is being used for CAD risk stratification. In patients with low to intermediate probability of acute coronary syndrome (ACS), it can be used as an initial test to rule out ACS owing to its high negative predictive value. It is also being utilized as an alternative to invasive coronary angiography in patients with equivocal stress test results.

Our case demonstrates a situation where CCTA proved to be a more accurate diagnostic tool than EKG stress testing. The results significantly altered management as the patient concluded his hospital stay with coronary artery bypass grafting (CABG). Alternatively, if CCTA was not performed and the cardiologist deemed the indeterminate stress test results as negative, the patient may have been discharged and may have had a deleterious cardiac outcome. Recent guidelines from the National Institute of Health and Care Excellence recommend CCTA as the initial diagnostic test in patients with suspected CAD [7]. However, contrast-related side effects, availability of test, and cost are the main barriers to this recommendation at this time. CCTA has also been shown to have limited diagnostic accuracy in patients with intracoronary stents. The PROMISE trial showed no significant difference in clinical outcomes of patients with suspected CAD who underwent anatomical stress testing with CCTA compared to those who underwent functional stress testing [8]. However it may be worthwhile to utilize CCTA as the initial CAD diagnostic test if no contraindications are noted.

## Conclusion

As a result of the availability of multiple noninvasive diagnostic tests with almost similar sensitivities for CAD, physicians often face this dilemma of choosing the right test for optimal evaluation of chest pain in patients with intermediate pretest probability of CAD. Optimal test selection requires an individualized patient approach. Our case describes a patient with intermediate probability of CAD presenting to the hospital with chest pain that resolved on admission and having a treadmill EKG stress test with indeterminate results. Decision to proceed with anatomical testing using CCTA was made and the patient was found to have significant CAD requiring CABG. Our experience with this case emphasizes the role of history taking, clinical judgement, and the risk/benefit ratio in deciding further workup when faced with inconclusive stress test results. Physicians should have a lower threshold for further workup of patients with inconclusive or even negative stress test results because of the diagnostic limitations of the test. Repeating the same test may result in uncertainty and indeterminate stress test should not be presumed as negative. Instead, utilizing a different, anatomical CAD test may be more valuable. Specifically, the case established the usefulness of CCTA in cases such as this where other CAD diagnostic testing is indeterminate.

## Data Availability

Data that supports this study have been referenced here.

## References

[1] Bourque JM, Beller GA. Value of exercise ECG for risk stratification in suspected or known CAD in the era of advanced imaging technologies. JACC Cardiovasc Imaging. 2015;8(11):1309–21. 10.1016/j.jcmg.2015.09.006 PMC4646721 26563861

[2] Christman MP, Bittencourt MS, Hulten E,* et al*. Yield of downstream tests after exercise treadmill testing. J Am Coll Cardiol. 2014;63(13):1264–74. 10.1016/j.jacc.2013.11.052. PMC4033667 24509269

[3] Alberto J, Román S, Vilacosta I,* et al*. Dipyridamole and dobutamine-atropine stress echocardiography in the diagnosis of coronary artery disease: comparison with exercise stress test, analysis of agreement, and impact of antianginal treatment. Chest. 1996;110(5):1248–54. 10.1378/chest.110.5.1248 8915229

[4] Previtali M, Lanzarini L, Fetiveau R,* et al*. Comparison of dobutamine stress echocardiography, dipyridamole stress echocardiography and exercise stress testing for diagnosis of coronary artery disease. Am J Cardiol. 1993;72(12):865–70. 10.1016/0002-9149(93)91097-2 8213540

[5] Bourque JM, Charlton GT, Holland BH, Belyea CM, Watson DD, Beller GA. Prognosis in patients achieving ≥10 METS on exercise stress testing: Was SPECT imaging useful? J Nucl Cardiol. 2011;18(2):230–7. 10.1007/s12350-010-9323-2. PMC3902109 21132417

[6] Duvall WL, Lane Duvall W, Savino JA,* et al*. Prospective evaluation of a new protocol for the provisional use of perfusion imaging with exercise stress testing. Eur J Nucl Med Mol Imaging. 2015;42(2):305–16. 10.1007/s00259-014-2864-x. 25367747

[7] Overview | Recent-onset chest pain of suspected cardiac origin: assessment and diagnosis | Guidance | NICE. https://www.nice.org.uk/guidance/cg95. Accessed 18 Apr 2020.

[8] Douglas PS, Hoffmann U, Patel MR,* et al*. Outcomes of anatomical versus functional testing for coronary artery disease. N Engl J Med. 2015;372(14):1291–300. 10.1056/NEJMoa1415516 PMC4473773 25773919

